# COVID-19 Impairs Immune Response to *Candida albicans*

**DOI:** 10.3389/fimmu.2021.640644

**Published:** 2021-02-26

**Authors:** Dominique Moser, Katharina Biere, Bing Han, Marion Hoerl, Gustav Schelling, Alexander Choukér, Tobias Woehrle

**Affiliations:** Department of Anesthesiology, LMU Hospital, Ludwig-Maximilians-University, Munich, Germany

**Keywords:** second hit, lipopolysaccharide, *Listeria monocytogenes*, yeast, fungal, SARS-CoV-2, acute respiratory distress syndrome

## Abstract

Infection with SARS-CoV-2 can lead to Coronavirus disease-2019 (COVID-19) and result in severe acute respiratory distress syndrome (ARDS). Recent reports indicate an increased rate of fungal coinfections during COVID-19. With incomplete understanding of the pathogenesis and without any causative therapy available, secondary infections may be detrimental to the prognosis. We monitored 11 COVID-19 patients with ARDS for their immune phenotype, plasma cytokines, and clinical parameters on the day of ICU admission and on day 4 and day 7 of their ICU stay. Whole blood stimulation assays with lipopolysaccharide (LPS), heat-killed *Listeria monocytogenes* (HKLM), *Aspergillus fumigatus*, and *Candida albicans* were used to mimic secondary infections, and changes in immune phenotype and cytokine release were assessed. COVID-19 patients displayed an immune phenotype characterized by increased HLA-DR^+^CD38^+^ and PD-1^+^ CD4^+^ and CD8^+^ T cells, and elevated CD8^+^CD244^+^ lymphocytes, compared to healthy controls. Monocyte activation markers and cytokines IL-6, IL-8, TNF, IL-10, and sIL2R*α* were elevated, corresponding to monocyte activation syndrome, while IL-1β levels were low. LPS, HKLM and *Aspergillus fumigatus* antigen stimulation provoked an immune response that did not differ between COVID-19 patients and healthy controls, while COVID-19 patients showed an attenuated monocyte CD80 upregulation and abrogated release of IL-6, TNF, IL-1α, and IL-1β toward *Candida albicans*. This study adds further detail to the characterization of the immune response in critically ill COVID-19 patients and hints at an increased susceptibility for *Candida albicans* infection.

## Introduction

The novel coronavirus 2019-nCov, also named SARS-CoV-2, has rapidly spread around the globe since its discovery in Wuhan, China, in December 2019. Infection with SARS-CoV-2 can lead to Coronavirus disease 2019 (COVID-19), where respiratory symptoms may include cough, breathing difficulties, and in severe cases, result in acute respiratory distress syndrome (ARDS) (1, 2). COVID-19 induces a plethora of immunological reactions affecting the innate and adaptive immune responses, which are critical to the clinical course and outcome of patients. Severe COVID-19 can result in excessive cytokine release or immunosuppression, with substantial morbidity and mortality mostly in older patient populations (1, 3–5).

While in the beginning of the COVID-19 pandemic, superinfections have been rarely reported (6), reports about secondary fungal infections as complications of severe COVID-19 are on the rise. In COVID-19 patients with ARDS, COVID-19 associated pulmonary aspergillosis (6–8) and COVID-19 associated candidiasis (9–14) have been described to complicate the clinical course. Although the exact pathogenesis of these coinfections remains unclear, there are several immunological mechanisms that can facilitate the development of fungal diseases. SARS-CoV-2 infection leads to the release of danger-associated molecular patterns. Consecutive activation of innate pattern recognition pathways may cause hyperinflammation during the antiviral immune response, leading to lung tissue damage and causing disruption of mucous membranes, thus contributing to an environment that allows for fungal infections (15–18). While most microbiological detection of *Candida* spp. as commensals does not have pathological relevance, transition into invasive candidiasis during critical illness has a reported lethality rate of up to 70% (19–21) and is considered a relevant complication in COVID-19 patients (15). Details regarding the susceptibility of these patients to *Candida* infection and characteristics of an impaired immune response have not been described yet.

In this study, 11 severe cases of COVID-19 with ARDS that required intubation and ICU treatment are characterized and compared to healthy controls. We detail the immune phenotype and cytokine release pattern of these patients on the day of admission, and on day 4 and day 7 of their ICU stay and present an *ex vivo* second hit model that reveals an impaired immune response against *Candida albicans*.

## Materials and Methods

### Study Subjects

Patients with pneumonia resulting from confirmed SARS-CoV-2 infection were included in this study upon admittance to the ICU, when they required intubation and ventilation. Patients with a malignant disease or previous immunosuppressive therapy were excluded. All patients received treatment according to the standard COVID-19 treatment protocol as of April 2020, including azithromycin for 5 days, and hydroxychlorquine for 7 days. Arterial blood was drawn on the day of ICU admission (day 0; d0) and on day 4 (d4) and day 7 (d7) of the ICU stay. The patients’ detailed clinical course was recorded electronically (QCare ICU, HIM GmbH, Bad Homburg, Germany). As controls, healthy volunteers were included.

### *Ex Vivo* Whole Blood Incubation Assay

Lithium heparin whole blood (300 µl) was diluted with an equal volume of RPMI 1640 (Sigma-Aldrich, Steinheim, Germany) and incubated with one of the following stimuli: Lipopolysaccharide (LPS; 10 µg/ml; Sigma-Aldrich, Steinheim, Germany), heat-killed *Listeria monocytogenes* (HKLM; 10^8^ cells/ml; InvivoGen Europe, Toulouse, France), *Aspergillus fumigatus* (*A. fumigatus*; 10 μg/ml; Raybiotech, Georgia, USA), *Candida albicans* lysate (*C. albicans*; 10 μg/ml; Allergopharma, Reinbeck, Germany), or vehicle only as negative control. Samples were incubated for 6 h at 37°C. Following incubation, supernatants were collected and stored at −80°C until cytokine measurement. The remaining sample was conserved with Transfix (Cytomark, Buckingham, UK) and stored at 4°C until flow cytometry analyses.

### Immune Phenotyping

For T cells, CD4^+^ or CD8^+^ cells were stained for co-expression of CD69, HLA-DR, CD38, PD-1, CD28, CD244 and CD226. Central memory T cells were detected by additional staining for CD62L, CD45RA, and CD45RO. For monocytes, cells were stained for co-expression of CD14 and CD16, CD69, CD40, CD80, CD86, TLR2 or HLA–DR. Except for TLR2 (Cat. No. 130-120-052, Miltenyi Biotec, Bergisch Gladbach, Germany), all antibodies were obtained from BD Biosciences (Cat. No. CD4: 555346, CD8: 557085, CD69-PE: 555531, HLA-DR: 559831, CD38: 345806, PD-1: 561273, CD28: 337181, CD244: 562350, CD226: 338314, CD62L: 555544, CD45RA: 550855, CD45RO: 560607, CD14: 345784, CD16: 338440, CD69-PerCp: 340548, CD40: 555589, CD80: 557227, CD86: 555665, Franklin Lakes, NJ, USA). Samples and antibodies were incubated for 20 min at room temperature, lysed for 10 min (BD FACS lysing solution, BD Biosciences Franklin Lakes, NJ, USA), washed, and analyzed by flow cytometry (Guava^®^ easyCyte™ 8HT Flow Cytometer, Merck Millipore, Billerica, MA, USA). For each measurement, 10,000 events were recorded. Data analysis was performed with InCyte Software for Guava^®^ easyCyte HT Systems (Merck Millipore, Billerica, MA, USA).

### Cytokine Measurements

Cytokine concentrations in plasma samples and *ex vivo* incubation assay supernatants were quantified using the MAGPIX Multiplexing System (Luminex, Austin, TX, USA) and custom-made Multiplex assays for detection of IFN*γ*, IL-1α, IL-1β, IL-2, IL-6, IL-8, IL-10, IL-18, TNF, and sIL2R*α*, according to the manufacturer’s instructions.

### Statistical Analyses

Data analysis was performed with commercially available software (SigmaPlot 12.5, Systat, Erkrath, Germany; GraphPad Prism 8.1.1, San Diego, CA, USA). Unless otherwise stated, results are expressed as median (IQR). For comparison between two groups, two-tailed unpaired Student’s *t*-test, Mann–Whitney-*U* test or Fisher’s exact test were used. Differences were considered significant at *P*<.05.

### Study Approval

Informed consent was obtained from next of kins or carers of all patients and from healthy volunteers, respectively. This study was performed after obtaining the LMU Medical Faculty ethics committee approval (#20-271 and #19-778) and conducted in agreement with the ethical norms and standards of the Declaration of Helsinki (22).

## Results

### Study Population

Between April and May 2020, 11 COVID-19 patients with ARDS were included in this study. The median age was 64 years, eight patients were male, and the median body mass index was 27.7 kg/m^2^. The median Horowitz index upon admission (day 0; d0) was 107 (d4: 182; d7: 199), indicating moderate ARDS (**Table 1**). Median duration for the requirement of ventilation was 15 days, with 23 days of ICU care and 34 days of hospitalization. In four out of 11 patients, blood sampling was discontinued before d4 due to extubation (n = 1), transfer to a different hospital (n = 1), or withdrawal of consent for further blood sampling for research purposes (n = 2). All patients were transferred to a rehabilitation hospital or into primary physician care, and no fatal outcome occurred during the evaluated time period.

**Table 1:** Characterization of COVID-19 patients and healthy controls.

Characteristic	COV	HC	*P* value
**Age (years)**	64 (57–72)	56 (47.5–63)	.062^a^
**Male sex (%)**	8 (72.7)	7 (77.8)	1.0^c^
**BMI (kg/m^2^)**	27.7 (25.1–31.1)	26.0 (23.9–29.9)	.209^b^
**PaO_2_/FiO_2_ ratio**			
day 0	107 (91–174)		
day 4	182 (138–190)		
day 7	199 (181–234)		
**Days**			
ventilated	15 (11.5–22.5)		
on ICU	23 (14.5–28)		
in hospital	34 (25.5–47)		
**Mortality, n (%)**			
on ICU	0 (0)		
at day 28	0 (0)		
**Leukocytes (cells/µl)**	10,600 (7,530–12,400)	5,550 (4,360–6,065)	**.005** ^a^
**Lymphocytes (%)**	11 (9–16)	28 (25–40)	**<.001** ^a^
**Lymphocytes (cells/µl)**	1,090 (764–1,316)	1,550 (1,185–2,180)	**.033** ^a^
**Monocytes (%)**	4 (3–7)	7 (6–9)	**.038** ^a^
**Monocytes (cells/µl)**	322 (202–628)	360 (320–465)	.732^a^
**Neutrophils (%)**	74 (64–82)	62 (49–66)	**.018** ^a^
**Neutrophils (cells/µl)**	7,530 (6,230–9,980)	2,980 (2,090–3,775)	**.010** ^a^
**Thrombocytes (×10^9^/L)**	336 (241–387)	211 (172–258)	**.006** ^b^
**Erythrocytes (×10^12^/L)**	4.01 (3.17–4.22)	4.93 (4.77–5.16)	**<.001** ^b^
**Hemoglobin (g/dl)**	11.5 (9.8–12.3)	15 (14.5–15.6)	**<.001** ^b^

Values are given as median (IQR). Differences between COV and HC were calculated using two-tailed unpaired Student’s t test (^a^), Mann Whitney U Test (^b^) or Fisher’s Exact test (^c^). COV, COVID-19, n = 11; HC, healthy controls, n = 9; ICU, intensive care unit.

Significant differences are indicated in bold font.

On ICU admission, COVID-19 patients presented with an increased leukocyte count due to elevated neutrophils, but with diminished lymphocyte count and lymphocyte and monocyte fractions. Thrombocytes were elevated but within the reference range, while erythrocytes and hemoglobin were reduced below the reference range, respectively. Characteristics of critically ill COVID-19 patients (COV) and healthy controls (HC) are summarized in **Table 1**.

### Immune State During Critical COVID-19

The activation state of T cells and monocytes was assessed in COV on the day of ICU admission (d0, n = 11) and in HC (n = 9), respectively. Time dependent changes were monitored by repeated analyses on d4 (n = 7) and d7 (n = 7) of the patients’ ICU stay.

#### CD4^+^ and CD8^+^ T Cell Phenotype

**Figure 1:**
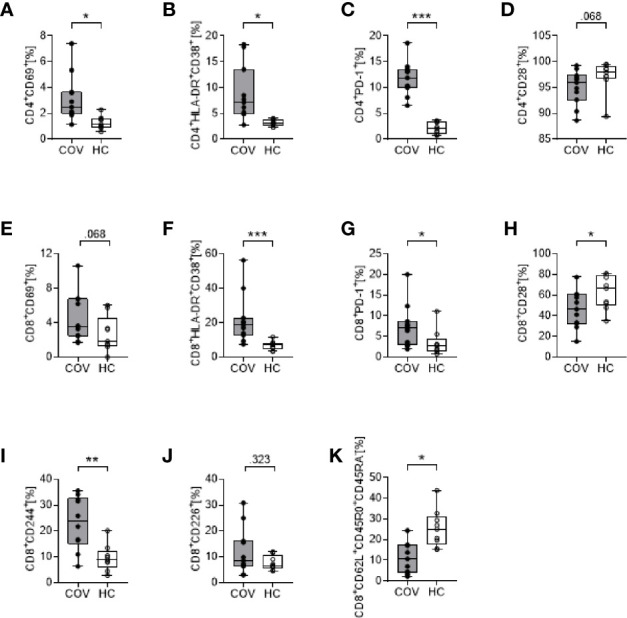
Cell surface marker expression on CD4^+^ T cells (upper row, **A–D**) and CD8^+^ T cells (lower rows, **E–K**) in COVID-19 patients (COV, n = 11, gray box) and healthy controls (HC, n = 9, white box). **(A)** CD4^+^CD69^+^, **(B)** CD4^+^HLA-DR^+^CD38^+^, **(C)** CD4^+^PD-1^+^, **(D)** CD4^+^CD28^+^, **(E)** CD8^+^CD69^+^, **(F)** CD8^+^HLA-DR^+^CD38^+^, **(G)** CD8^+^PD-1^+^, **(H)** CD8^+^CD28^+^, **(I)** CD8^+^CD244^+^, **(J)** CD8^+^CD226^+^, **(K)** CD8^+^CD62L^+^CD45RO^+^CD45RA^-^. Values represent percentages of surface marker positive cells of total CD4^+^ or CD8^+^ cells. Boxes indicate median and interquartile range; whiskers represent minimum and maximum, dots represent single values. Differences between groups were calculated using unpaired two-tailed *t* test or Mann–Whitney *U* test, *P < .05, **P < .01, ***P < .001.

In COV, we detected a pronounced activation of CD4^+^ and CD8^+^ T cells with significantly augmented proportions of CD69^+^, HLA-DR^+^CD38^+^ and PD-1^+^ cells (**Figures 1A–C, E–G**). In addition, CD8^+^ but not CD4^+^ T cells showed a reduced surface expression of CD28 (**Figures 1D, H**), high levels of the cytotoxic cell marker molecule CD244, and increased CD226 expression (**Figures 1I, J**). The percentage of CD8^+^CD62L^+^CD45R0^+^CD45RA^−^ central memory T cells showed a pronounced reduction in COV compared to HC (**Figure 1K**), while CD4^+^CD62L^+^CD45R0^+^CD45RA^−^ did not differ (not shown).

Over time (d4, d7), the percentage of HLA-DR^+^CD38^+^CD4^+^ and HLA-DR^+^CD38^+^CD8^+^ T cells increased, reaching statistical significance on d7, compared to d0 (CD4^+^ T cells d0: 8.83 ± 5.34, d7: 16.24 ± 5.69, *P* = .013; CD8^+^ T cells d0: 21.71 ± 14.35, d7: 33.57 ± 23.71, *P* = .043). All other surface markers remained unchanged over time (d4, d7; data not shown).

#### Monocyte Phenotype and Subsets

**Figure 2:**
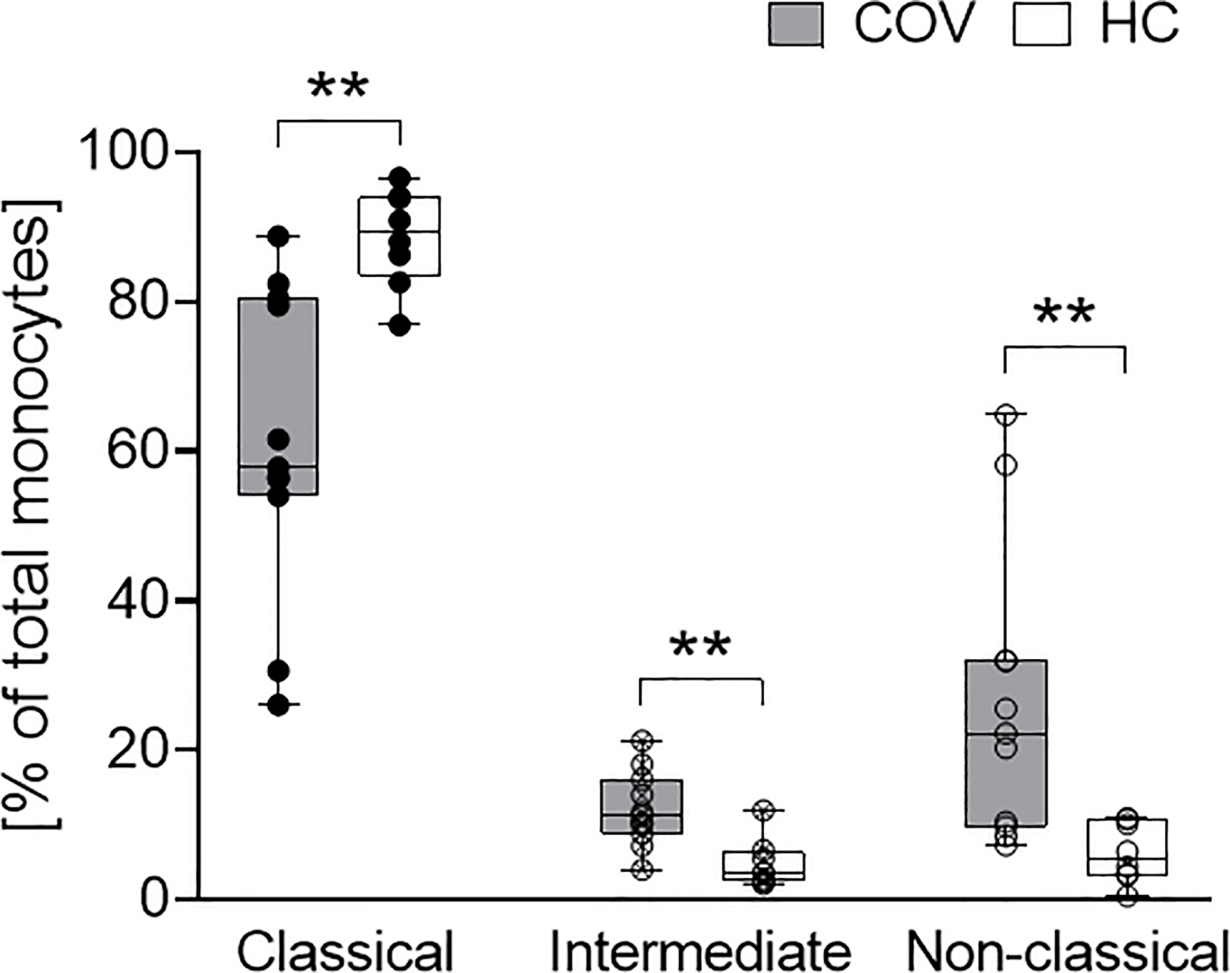
Classical (CD14^++^CD16^-^, left), intermediate (CD14^++^CD16^+^, middle) and non-classical (CD14^+^CD16^++^, right) monocyte subsets in COVID-19 patients (COV, n = 11, gray boxes) and healthy controls (HC, n = 9, white boxes). Values represent percentages of total monocytes. Boxes indicate median and interquartile range; whiskers represent minimum and maximum, dots represent single values. COV samples were compared to HC samples using unpaired two-tailed *t* test or Mann–Whitney *U* test, **P < .01.

Activation markers CD69, CD40, CD80, CD86, TLR2 and HLA-DR on monocytes were elevated in COV compared to HC, with significant differences for CD86 and TLR2 (**Table 2**). In COV, the proportion of classical monocytes (CD14^++^CD16^−^) was diminished compared to HC, while intermediate (CD14^++^CD16^+^) and non-classical (CD14^+^CD16^++^) subtypes showed a significant enrichment (**Figure 2**). Monocyte activation markers and monocyte subset patterns did not change on d4 and d7 (data not shown).

**Table 2:** Cell surface marker expression on monocytes of COVID-19 patients and healthy controls.

Surface marker Monocytes	COV	HC	*P* value
**CD14^+^CD69^+^**	4.96 (3.79 – 7.05)	1.94 (1.09 – 5.80)	.058^b^
**CD14^+^CD40^+^**	4.62 (2.86–8.11)	2.12 (0.14–4.94)	.068^b^
**CD14^+^CD80^+^**	3.69 (1.27–5.61)	1.83 (0.51–3.37)	.111^b^
**CD14^+^CD86^+^**	46.43 (28.93–68.46)	21.86 (9.73–35.83)	**.014^a^**
**CD14^+^TLR2^+^**	97.92 (95.82–99.06)	91.44 (88.99–93.46)	**<.001^a^**
**CD14^+^HLA-DR^+^**	71.24 (64.45–92.18)	75.07 (55.25–85.44)	.440^a^

Values are given as median (IQR) and represent percentages of surface marker positive cells of total monocytes. COV, COVID-19 (n = 11); HC, healthy controls (n = 9). Differences between COV and HC were calculated using two-tailed unpaired Student’s t test (^a^) or Mann–Whitney U Test (^b^).

Significant differences are indicated in bold font.

#### Plasma Cytokine Pattern

**Figure 3:**
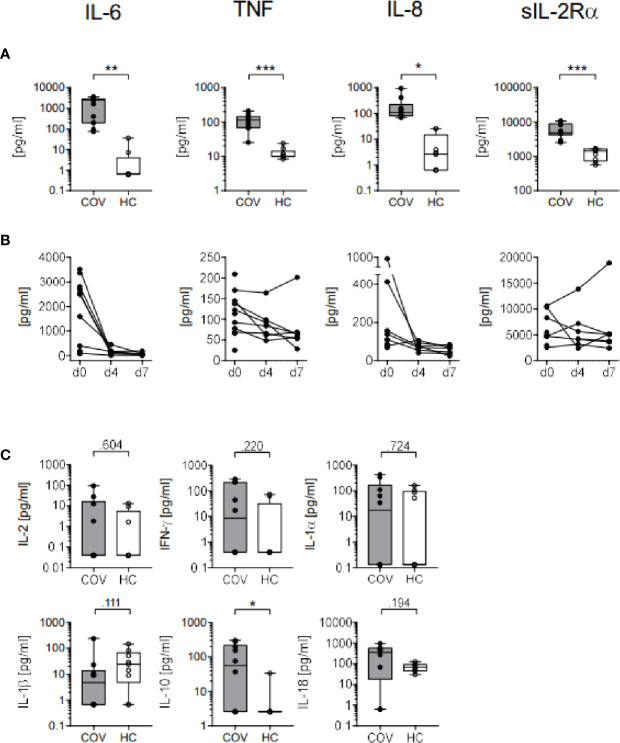
Plasma cytokine concentrations in COVID-19 patients (COV, n=10, gray box) and healthy controls (HC, n = 9, white box). **(A)** IL-6, TNF, IL-8 and sIL2Rα cytokine concentrations (pg/ml) on day 0 and **(B)** on days (d) 0, 4 (n = 7), and 7 (n = 7) in COV. **(C)** Concentrations of IL-2, IFNγ, IL-1α, IL-1β, IL-10, and IL-18 on day 0 (COV, n = 8–11) and healthy controls (HC, n = 9, white box). Boxes indicate median and interquartile range, whiskers represent minimum and maximum, dots represent single values. Statistical differences between COV and HC were calculated using unpaired two-tailed *t* test or Mann–Whitney *U* test, *P < .05; **P < .01; ***P < .001.

Upon ICU admission, concentrations of IL-6, IL-8, TNF and sIL2R*α* (**Figure 3A**) were significantly increased, indicating monocyte activation syndrome in these patients. Over time, levels of IL-6, IL-8, TNF and sIL2R*α* decreased from d0 to d4 and remained low on d7 (**Figure 3B**). Additionally, plasma cytokine concentrations of IL-10 were found elevated in COV, and IFN*γ* and IL-18 tended to be higher than in HC while IL-2, IL-1α and IL-1β did not differ (**Figure 3C**).

### *Ex Vivo* Immune Response in a Second Hit Model

Bacteria and fungi can cause secondary infections and worsen the patient’s prognosis (20, 23). Thus, we aimed to assess the immune response against the Gram-negative bacterial component LPS, the Gram-positive bacteria *L. monocytogenes* and the fungi *C. albicans* and *A. fumigatus* by *ex vivo* incubation of whole blood with the respective antigens to mimic microbial superinfection in critically ill COVID-19 patients. After stimulation, changes in cytokine release and leukocyte phenotype in COV and HC were determined.

#### Cytokine Pattern After Antigen Stimulation

**Figure 4:**
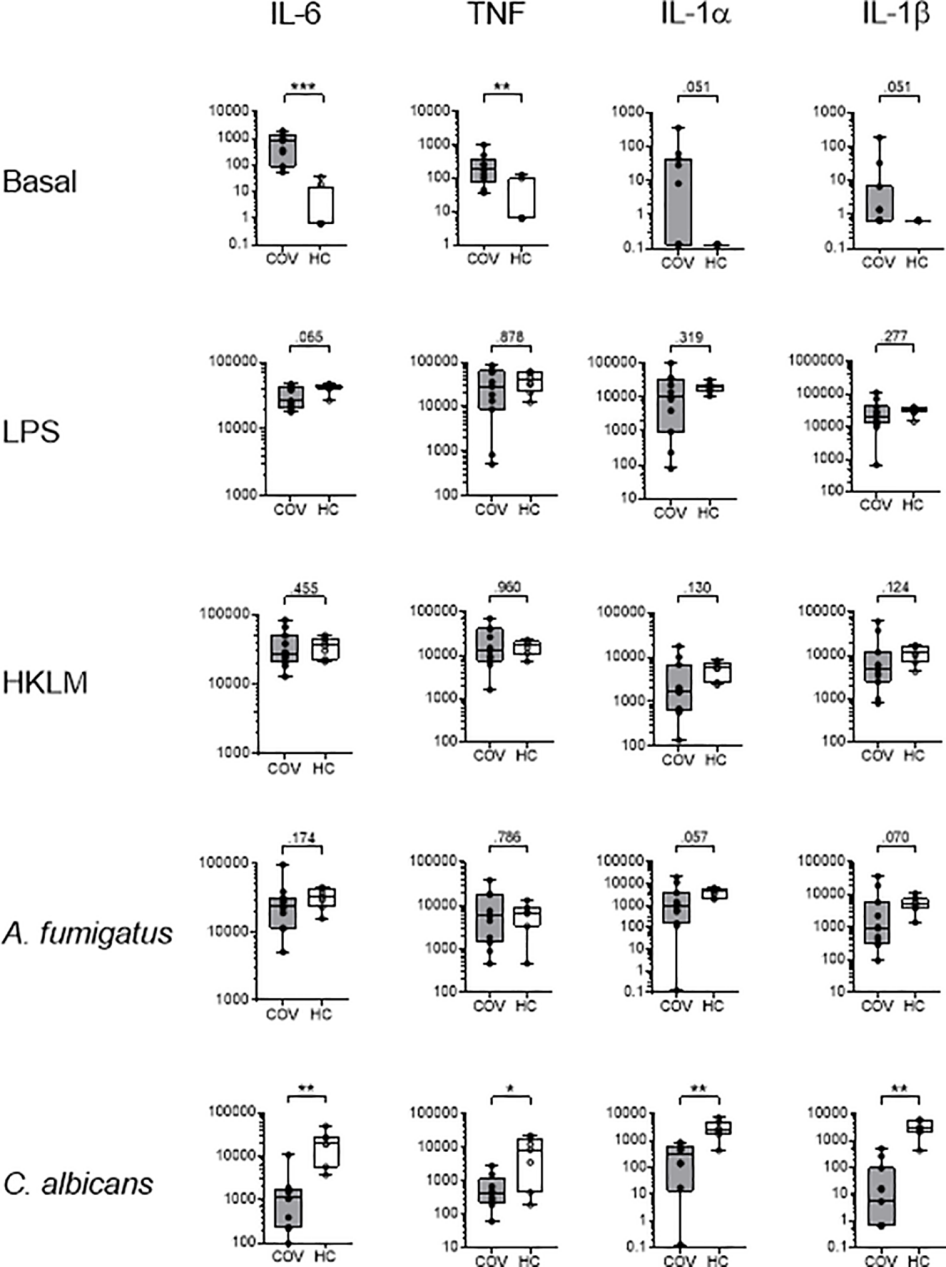
Cytokine concentrations (pg/ml) of IL-6, TNF, IL-1α and IL-1β in supernatants of whole blood samples from COVID-19 patients (COV, n=11, gray box) and healthy controls (HC, n=7-9, white box) after *ex vivo* antigen incubation (basal, LPS, HKLM, *A. fumigatus*, *C. albicans*). Boxes indicate median and interquartile range, whiskers represent minimum and maximum, dots represent single values. Statistical differences were calculated using unpaired two-tailed *t* test or Mann Whitney *U* test, *P < .05, **P < .01, ***P < .001.

Cytokine patterns in basal control samples were similar to those of native blood, with elevated cytokine levels in COV, compared to HC. Stimulation with LPS, HKLM and *A. fumigatus* induced comparable increases in concentrations of IL-6, TNF, IL-1α, IL-1β, IL-8 and IL-10 in COV and HC, while an IFN*γ* response was only observed in HC, and IL-2, sIL2R*α*, and IL-18 were unaffected in both groups, compared to basal controls (**Figure 4** and **Table 3**). Similarly, *C. albicans* induced an increase of IL-6, TNF, IL-1α, IL-1β, IFN*γ*, IL-10 and IL-8 in HC; however, the release of IL-6, TNF, IL-1α, IL-1β, IL-10, and IFN*γ* was abrogated in COV, with no statistical difference of IL-10, IL-6, TNF, IL-1β, and IFN*γ* compared to basal controls (**Figure 4** and **Table 3**).

**Table 3:** Cytokine concentrations in supernatants of COVID-19 patients and healthy controls after *ex vivo* antigen incubation (basal, LPS, HKLM, *A. fumigatus*, *C. albicans*).

Cytokine (pg/ml)	Basal			LPS			HKLM			
	COV	HC	*P*	COV	HC	*P*	COV	HC	*P*	
**IL-2**	0.04 (0.04–0.70)	< LOD	.112^b^	0.04 (0.04–0.70)	< LOD	.112^b^	0.04 (0.04–1.31)	< LOD	.112^b^	
**IFN*γ***	0.40 (0.4–111.20)	< LOD	.093^b^	124.52 (0.40–166.20)	3189.40 (1385.29–6144.24)	**<.001** ^b^	10.82 (0.40–134.48)	453.41 (261.92–619.87)	**<.001** ^b^	
**IL-10**	2.60 (2.6–44.13)	< LOD	.073^b^	1282.71 (290.88–2492.44)	488.62 (282.72–591.88)	.124^b^	587.83 (406.43–2625.29)	635.23 (438.27–35.63)	.717^b^	
**IL-8**	1379.80 (378.80–2719.66)	218.18 (85.07–388.38)	**.011** ^b^	5513.64 (3257.59–8002.47)	3690.49 (2237.11–8048.28)	.610^a^	5144.01 (2633.66–8234.36)	5495.46 (2232.60–12744.94)	.717^b^	
**sIL2Rα**	2733.10 (1877.33–4326.63)	542.45 (374.07–681.67)	**<.001** ^b^	2938.69 (1602.30–769.40)	537.01 (409.95–617.38)	**<.001** ^b^	3081.62 (1618.11–4023.78)	565.92 (379.75–604.33)	**<.001** ^b^	
**IL-18**	224.95 (32.96–476.05)	0.64 (0.64–31.99)	**.006** ^b^	300.65 (108.85–616.43)	0.64 (0.64–76.99)	**.001** ^b^	277.82 (107.24–490.20)	0.64 (0.64–11.52)	**<.001** ^b^	
**Cytokine (pg/ml)**	***A. fumigatus***			***C. albicans***					
	**COV**	**HC**	***P***	**COV**	**HC**	***P***			
**IL-2**	0.04 (0.04–0.24)	< LOD	.112^b^	0.04 (0.04–4.89)	< LOD	.563^b^			
**IFN**–	10.36 (0.40–102.27)	306.12 (174.10–462.95)	**<.001** ^b^	2.59 (0.40–54.88)	206.30 (43.28–418.47)	**.022** ^b^			
**IL-10**	246.74 (132.65–952.70)	196.50 (162.39–466.64)	.526^b^	2.60 (2.60–44.92)	292.05 (166.25–408.86)	**.002** ^b^			
**IL-8**	3520.71 (1909.91–7727.71)	3032.29 (1399.32–6602.88)	.415^b^	2745.24 (2252.91–4110.54)	2290.30 (1990.57–4721.37)	.464^b^			
**sIL2Rα**	3017.12 (1762.30–3859.42)	549.70 (362.38–628.19)	**<.001** ^b^	3091.67 (1905.49–4110.20)	529.74 (414.47–615.01)	**<.001** ^b^			
**IL-18**	233.42 (140.17–442.54)	0.64 (0.64–0.64)	**<.001** ^b^	139.78 (18.82–443.98)	0.64 (0.64–11.11)	**.004** ^b^			

Concentrations (pg/ml) are given as median (IQR). COV, COVID-19 (n = 9–11); HC, healthy controls (n = 7–9); LOD, Limit of detection. Differences between COV and HC were calculated using unpaired Student’s t test (^a^) or Mann–Whitney U test (^b^).

Significant differences are indicated in bold font.

#### T Cell and Monocyte Phenotype After Antigen Stimulation

Since COV showed a weakened cytokine response to *C. albicans* but not to LPS, HKLM, or *A. fumigatus*, we exemplarily compared T cell and monocyte phenotypes of *C. albicans* and HKLM stimulated samples of COV and HC.

In T cells, COV and HC displayed a similar expression pattern of CD69, HLA-DR/CD38, PD-1, CD28, CD244, and CD226 on both CD4^+^ and CD8^+^ T cells after *C. albicans* or HKLM stimulation compared to basal control (**Supplementary Table 1**).

**Figure 5:**
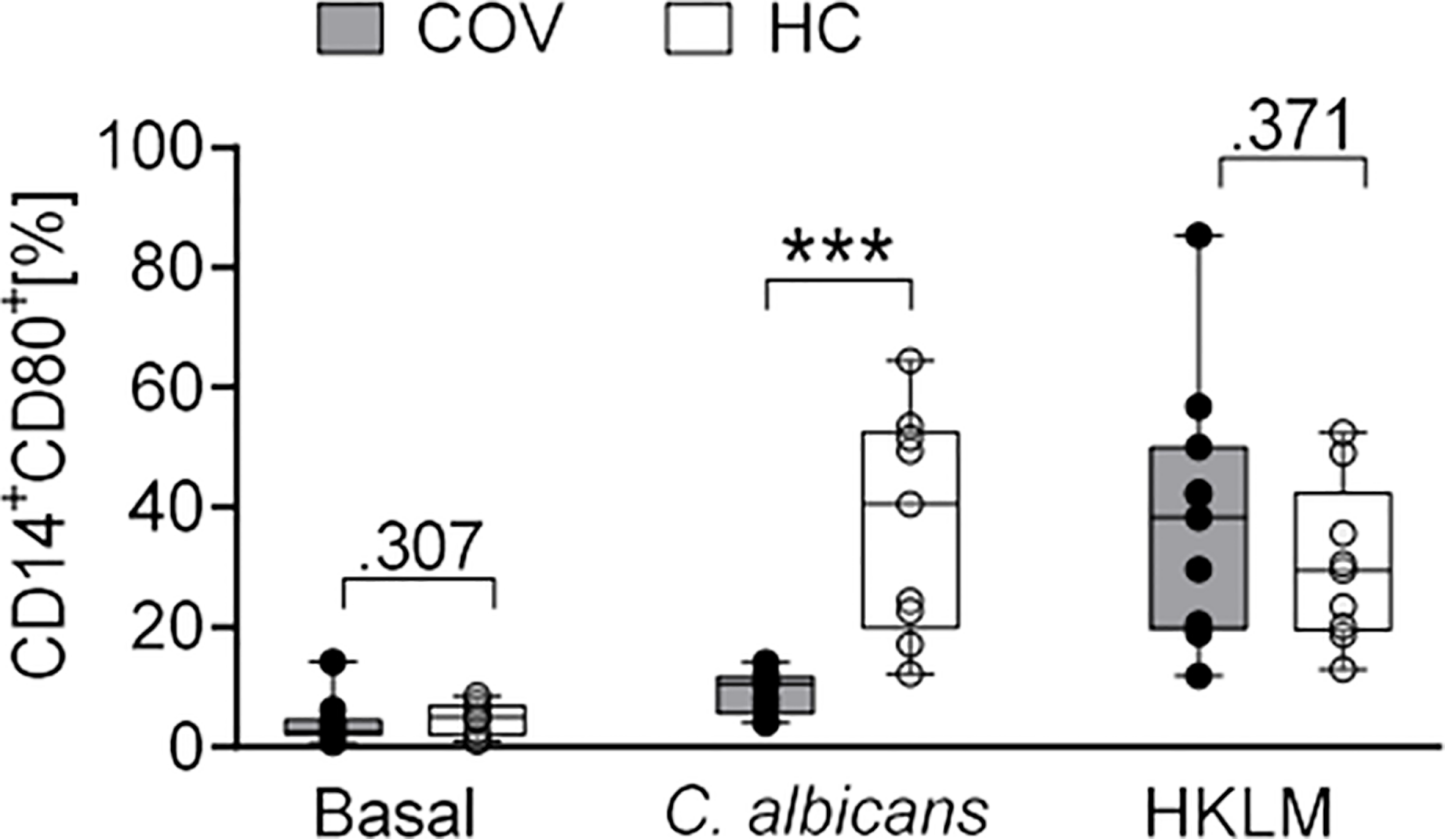
CD80 expression on monocytes after *ex vivo* antigen incubation (basal, *C. albicans*, HKLM) in COVID-19 patients (COV, n = 11, gray box) and healthy controls (HC, n = 9, white box). Values represent percentages of surface marker positive cells of total CD14^+^ cells. Boxes indicate median and interquartile range; whiskers represent minimum and maximum, dots represent single values. Differences between groups were calculated using unpaired two-tailed *t* test or Mann–Whitney *U* test, ***P < .001.

Similarly, COV monocyte subsets of classical, intermediate, and non-classical monocytes showed no differences after stimulation (**Supplementary Figure 1**), and expression of CD69, CD40, CD86, TLR2, and HLA-DR did not differ between antigens or COV and HC (**Supplementary Table 2**). In contrast, expression of CD80 was significantly lower after *C. albicans* stimulation in COV compared to HC (**Figure 5**).

Taken together, critically ill COVID-19 patients and healthy controls did not substantially differ in their phenotype and cytokine response to LPS, HKLM and *A. fumigatus*, but a lower expression of CD80 on monocytes and an abrogated release of IL-10, IL-6, TNF, IL-1α, and IL-1β indicates an impaired response to *C. albicans*.

## Discussion

In the present study, we provide detailed analyses of the immune state of critically ill COVID-19 patients during their ICU stay and compare them to healthy controls. In COV, low lymphocyte counts are combined with pronounced T cell activation with elevated expression of CD69, HLA-DR/CD38 and PD-1, while monocytes display highly proinflammatory properties. An *ex vivo* whole blood assay reveals a similar immune response of COV and HC to LPS, HKLM and *A. fumigatus*, but an impaired response of COV toward *C. albicans*.

### T Cell and Monocyte Phenotypes and Plasma Cytokines in Severe COVID-19

Both CD4^+^ and CD8^+^ T cells in COV showed an increased surface expression of CD69 and co-expression of HLA-DR and CD38, a constellation which has been characterized as T cell hyperactivity (24–26). T cell hyperactivation in COVID-19 is in accordance with findings from other groups (24, 27). High proportions of CD226^+^ and CD244^+^ CD8^+^ T cells correspond to a described virus-specificity with increased cytotoxic effector function (28–31). By exhibiting high proliferative and cytotoxic effector functions (25), these cells contribute to viral control (32). Concomitantly, T cells expressed high levels of PD-1, which is involved in T cell inhibition (33). Elevated PD-1 expression on CD4^+^ and CD8^+^ T cells is found on activated T cells and has been recognized as an indicator for cell exhaustion during chronic human viral infections (34, 35) and in COVID-19 (24, 36, 37).

Persistent viral antigen exposure during chronic viral infections such as HIV was found to induce a terminal differentiation into effector type over memory type CD8^+^ T cells, which ultimately experience immune exhaustion and succumb to AICD before pathogen eradication (25). The pathomechanism of SARS-CoV-2 infection differs from chronic HIV or HCV infection, however, given the reduced proportion of central memory CD8^+^ T cells in COV, it may be speculated that prolonged SARS-CoV-2 exposure leads to excessive T cell activation, where terminal differentiation into effector cells predominates memory cell development. This can be followed by cell exhaustion and AICD, resulting in lymphopenia and thus, may be relevant for the outcome of COVID-19 (38).

Monocytes of COV displayed in parts an enhanced expression of activation markers, indicating an increased potential to activate the adaptive immune response. Unaffected expression of HLA-DR in COV is in contrast to previous reports of downregulated HLA-DR in COVID-19 (39–41). However, the immune response of COVID-19 patients with ARDS can be categorized into three different groups: i) immune dysregulation with low HLA-DR expression and features of sepsis-induced immune paralysis, ii) monocyte activation syndrome with high inflammatory responses, mildly affected HLA-DR levels and sustained immune response and iii) an intermediate functional state, lacking these immune dysregulations (40). Here, the observed mildly affected monocyte HLA-DR expression, the clear increase of plasma cytokines and the pronounced immune response toward LPS, HKLM and *A. fumigatus* in stimulation assays infers monocyte activation syndrome rather than immune paralysis (40, 42). Accordingly, monocyte subsets in COV show a high non-classical proportion, confirming previous reports (41). Except for changes in HLA-DR^+^CD38^+^ co-expression on T cells, there were no further alterations in surface marker expression neither on T cells nor on monocytes over time, indicating persistence of SARS-CoV-2-induced immunological alterations.

Cytokine release patterns further confirmed monocyte activation syndrome in COV (40, 42). IL-6 and IL-8 plasma concentration declined on d4 and d7, which may in parts be caused by the anti-inflammatory properties of hydroxychloroquine and azithromycin, which both possess IL-6 lowering activity (43, 44). Similarly and in accordance with previous reports (42, 45), the median IL-18 concentration was increased in COV, without statistical significance. In contrast, IL-1β remained low, suggesting disturbances in inflammasome activation during COVID-19.

### *Ex Vivo* Immune Stimulation Assays Uncover Deficiency Toward *C. albicans *in COVID-19

Fungal superinfections have been observed in COVID-19, with COVID-19-associated candidiasis representing one major complication (12, 15, 46–48). The involvement of immune dysfunction in *Candida* spp. superinfections during COVID-19 is not yet understood (15). Thus, leukocyte phenotypes and cytokine responses were investigated by whole blood *ex vivo* incubation assays with LPS, HKLM, *A. fumigatus*, and *C. albicans*. The commensal polymorphic fungus *C. albicans* represents a member of the human microbiome and does not harm the host in an immunocompetent state. During immune system disturbances however, it can cause infections of superficial skin and mucous membranes up to life-threatening systemic infections (12, 49–51).

In T cells and monocytes, activation capacities as well as co-stimulatory and antigen-presenting properties in monocytes were maintained, and in contrast to HC, stimulation did not influence monocyte subtype proportions in COV. However, monocyte surface expression of co-stimulatory CD80 in response to *C. albicans* was significantly less increased compared to HC, indicating attenuated monocyte activation by this fungus and as a consequence, reduced co-stimulatory effects of CD80 on the adaptive immune response. This conclusion is supported by the impaired cytokine release profile. Here, concentrations of nearly all tested monocyte activation syndrome relevant cytokines were unaffected by *C. albicans* stimulation, whereas LPS-, HKLM- and *A. fumigatus*-induced cytokine secretion was similar to HC. The lacking immune response toward *C. albicans* antigen but not toward HKLM, which can both act as TLR2 agonists (21, 52, 53), or toward LPS or *A. fumigatus* adds to the notion of preserved immune function in COV, and is inconsistent with results indicating a generally attenuated TLR response of the peripheral innate immune system from COVID-19 patients (54).

An adequate immune response against *C. albicans* is elicited after sensing of fungal cell wall components such as *β*-glucans and *α*-mannans by distinct surface pattern recognition receptors on monocytes and macrophages. These include in particular C-type lectin receptors (*e.g*., Dectin-1, Dectin 2/3, Mincle), NOD-like receptors and TLR2/4 (55–57). Activation of these receptors induces activation of downstream Syk and NF-*κ*B signaling, resulting in NLRP3 inflammasome activation and production of pro-inflammatory cytokines such as TNF, IL-1β and IL-18, which mediate fungicidal activity (55, 57, 58). Although we observed enhanced IL-18 concentrations in COV, a SARS-CoV-2-induced activation of NLRP3 as described by others (59, 60) is in discordance with low IL-1β levels and the lack of IL-1β increase after stimulation with *C. albicans*. To elucidate the exact mechanism of the disturbed immune response to *C. albicans* on the monocyte level, further investigations are needed, such as functional analyses of relevant surface receptors like Dectin-1 and -2 (57) and the associated Syk signaling pathway (61) as well as the pyroptotic signaling pathway, containing NLRP3 inflammasome and caspase-1 activation and IL-1β secretion (58).

Limitations of this work include the small sample size, which can only partially be compensated for by analyses of multiple time points. Due to a lack of patients with pneumonia caused by other viruses than SARS-CoV-2, healthy volunteers were used as controls for our comparative analyses.

Despite these limitations, our results provide first evidence of a disturbed immune response toward *C. albicans*, which may hint at an increased susceptibility toward infection with *C. albicans* in critically ill COVID-19 patients. We consider the immune response characterization of critical COVID-19 cases as relevant for the field, and the blunted cytokine response to stimulation with *C. albicans* noteworthy to both immunologists and clinicians.

## Data Availability Statement

The raw data supporting the conclusions of this article will be made available by the authors, without undue reservation.

## Ethics Statement

The studies involving human participants were reviewed and approved by the Ludwig-Maximilians University Medical Faculty Ethics Committee. The patients/participants or next of kins/carers provided their written informed consent to participate in this study.

## Author Contributions

DM, AC, and TW designed the study. DM, KB, BH, MH, and TW performed experiments and collected data, and data analysis was performed by DM and TW. The article was drafted by DM, AC, and TW, and critical revision for important intellectual content was performed by all authors (DM, KB, BH, MH, GS, AC, and TW). All authors contributed to the article and approved the submitted version.

## Funding

This work was supported by the German Aerospace Center (DLR) and the Federal Ministry of Economic Affairs and Technology [50WB1931, RP1920].

## Conflict of Interest

The authors declare that the research was conducted in the absence of any commercial or financial relationships that could be construed as a potential conflict of interest.

## Abbreviations

AICD, activation-induced cell death; ARDS, acute respiratory distress syndrome; COV, COVID-19 cohort; COVID-19, coronavirus disease-2019; danger-associated molecular patterns; HC, healthy control cohort; HKLM, heat-killed *Listeria monocytogenes;* ICU, intensive care unit.
