# Supplementary material for: COVID-19 Impairs Immune Response to Candida albicans
Source: Front Immunol. 2021 Feb 26;12:640644. doi: 10.3389/fimmu.2021.640644 (PMC7953065; doi:10.3389/fimmu.2021.640644)
Supplement: Supplementary file 1 [file DataSheet_1.pdf]

Moser et al., Supplementary material

Supplementary Table 1. Activation marker expression on CD4$^+$ and CD8$^+$ T cells of COVID-19 patients and healthy controls after *ex vivo* antigen incubation (basal, *C. albicans*, HKLM).

**Surface marker**	**Basal**			***C. albicans***			**HKLM**		
	**COV**	**HC**	***P***	**COV**	**HC**	***P***	**COV**	**HC**	***P***
**CD4$^+$ T cells**									
CD69	2.68 (2.31 – 4.57)	1.14 (0.65 – 1.90)	**.002**[b]	6.06 (2.15 – 7.19)	2.83 (1.58 – 6.44)	.281[a]	5.24 (2.29 – 9.86)	3.31 (2.38 – 4.89)	.174[a]
HLA-DR/CD38	5.69 (4.13 – 14.12)	2.20 (1.89 – 4.22)	**.002**[b]	6.17 (4.44 – 14.58)	3.45 (2.48 – 4.24)	**.002**[b]	7.14 (4.49 – 12.71)	3.33 (1.58 – 3.70)	**<.001**[b]
PD-1	7.87 (6.11 – 11.39)	2.03 (1.13 – 3.20)	**<.001**[b]	10.12 (7.88 – 14.49)	2.58 (2.01 – 4.33)	**.003**[b]	10.70 (8.31 – 16.40)	2.82 (1.76 – 4.37)	**.002**[b]
CD28	97.18 (95.73 – 98.76)	98.71 (97.88 – 99.07)	.068[b]	96.22 (93.27 – 98.03)	98.57 (96.59 – 98.98)	.111[b]	97.11 (95.09 – 98.16)	98.50 (97.38 – 98.88)	.171[b]
**CD8$^+$ T cells**									
CD69	5.19 (2.64 – 7.53)	2.87 (1.30 – 5.10)	.099[a]	7.38 (5.11 – 10.22)	8.11 (5.31 – 17.36)	.348[b]	10.20 (9.04 – 13.24)	12.57 (5.79 – 18.47)	.362[b]
HLA-DR/CD38	18.01 (16.05 – 20.10)	4.13 (3.34 – 6.61)	**<.001**[b]	16.65 (13.36 – 26.68)	6.75 (3.16 – 10.27)	**.002**[a]	17.41 (12.11 – 22.12)	6.70 (3.350 – 10.07)	**.001**[b]
PD-1	3.21 (1.40 – 4.37)	2.73 (1.59 – 4.94)	1.00[b]	6.76 (3.21 – 8.32)	4.93 (2.46 – 9.26)	.902[b]	6.02 (4.48 – 6.89)	3.48 (2.32 – 8.82)	.599[a]
CD28	46.52 (28.16 – 65.33)	66.48 (47.87 – 72.35)	.081[a]	41.32 (31.07 – 57.39)	58.15 (44.69 – 68.37)	.140[a]	49.15 (28.73 – 54.80)	60.34 (44.44 – 71.12)	.124[a]
CD244	8.29 (5.13 – 13.38)	2.67 (1.71 – 4.63)	**<.001**[b]	7.52 (6.52 – 15.72)	5.17 (2.83 – 6.03)	**.032**[a]	11.67 (6.99 – 16.36)	3.93 (2.30 – 8.30)	**.005**[b]
CD226	9.09 (5.29 – 12.06)	2.13 (1.15 – 4.77)	**.001**[b]	5.67 (4.78 – 12.11)	3.73 (2.02 – 7.59)	.178[b]	11.54 (5.06 – 17.09)	4.44 (2.31 – 10.80)	.078[a]

Values are given as median (IQR) and represent percentages of surface marker positive cells of total CD4$^+$ or CD8$^+$ cells. COV, COVID-19 (n=11); HC, healthy controls (n=9). Differences between COV and HC were calculated using two-tailed unpaired student´t t-test ([a]) or Mann-Whitney-U Test ([b]).

Supplementary Table 2. Cell surface marker expression on monocytes of COVID-19 patients and healthy controls after *ex vivo* antigen incubation (basal, *C. albicans*, HKLM).

Surface marker	Basal			*C. albicans*			HKLM		
	**COV**	**HC**	**P value**	**COV**	**HC**	**P value**	**COV**	**HC**	**P value**
**CD69**	4.95 (2.71 – 10.65)	2.98 (2.44 – 4.64)	.224[b]	6.51 (4.66 – 8.12)	7.44 (4.31 – 8.63)	.775[b]	9.44 (5.90 – 16.92)	12.11 (8.26 – 14.67)	.783[a]
**CD40**	4.34 (3.96 – 7.60)	3.76 (2.53 – 5.04)	.164[a]	8.35 (3.87 – 11.34)	6.26 (2.35 – 15.67)	.653[b]	15.69 (8.80 – 23.78)	12.40 (4.89 – 13.97)	.079[a]
**CD86**	52.85 (40.86 – 70.01)	60.94 (40.94 – 76.42)	.544[a]	68.91 (55.99 – 79.96)	81.39 (53.40 – 92.10)	.531[a]	70.67 (63.01 – 80.51)	65.80 (46.53 – 81.70)	.287[b]
**TLR2**	98.70 (94.26 – 99.27)	95.82 (91.96 – 97.47)	.129[b]	97.55 (89.05 – 98.97)	89.84 (85.43 – 95.30)	.091[a]	95.43 (88.89 – 98.25)	83.52 (81.73 – 88.33)	**<.001**[a]
**HLA-DR**	78.90 (72.33 – 89.46)	88.05 (79.68 – 89.30)	.425[b]	88.26 (82.35 – 93.80)	75.14 (42.27 – 84.91)	**.013**[b]	87.06 (71.94 – 92.57)	53.67 (36.93 – 72.79)	**.005**[b]

Values are given as median (IQR). COV, COVID-19 (n=11); HC, healthy controls (n=8-9). Differences between COV and HC were calculated using two-tailed unpaired student´t t-test ([a]) or Mann-Whitney-U Test ([b]).

Supplementary Figure 1

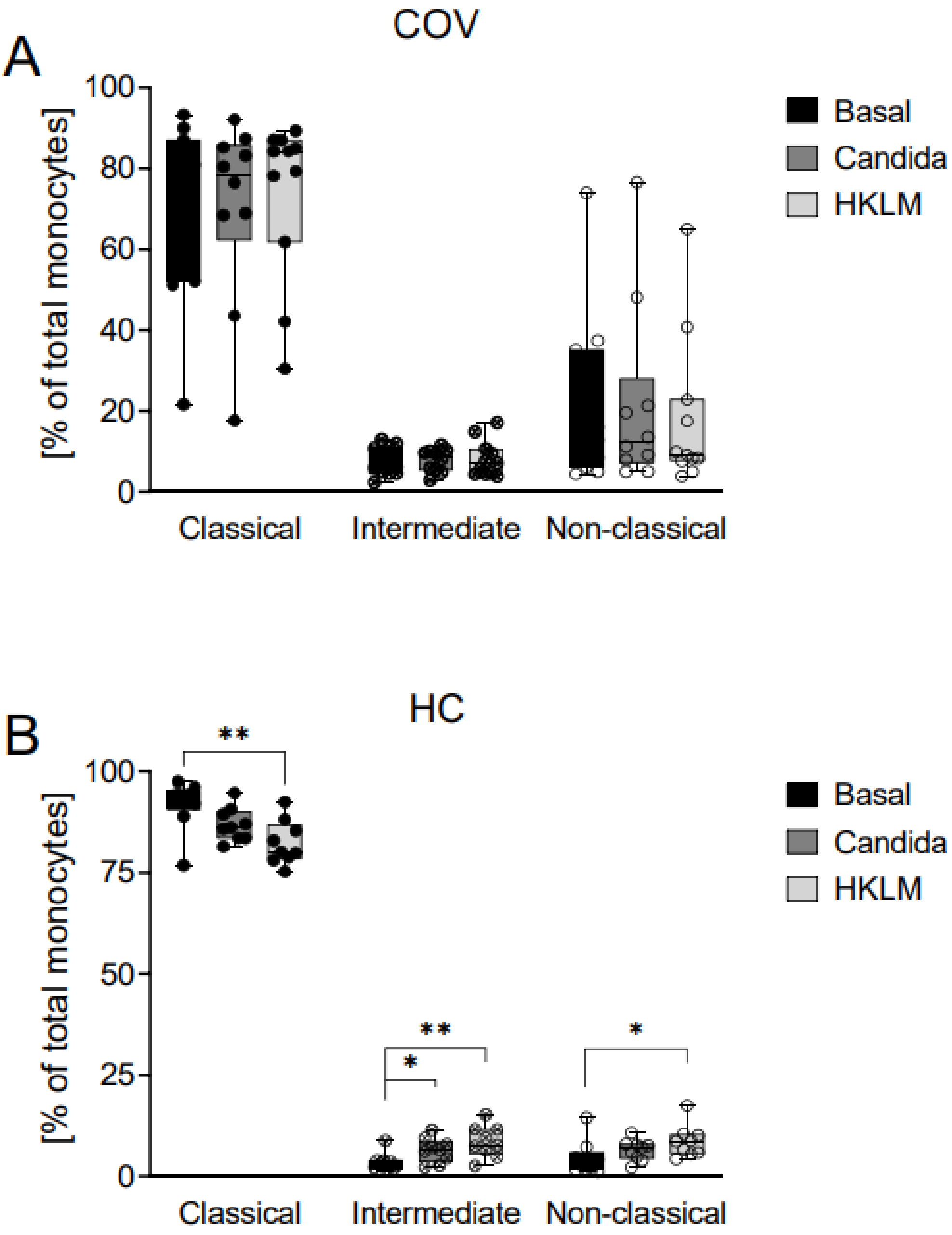

Supplementary Figure 1. Classical (CD14++CD16-, left), intermediate (CD14++CD16+, middle) and non-classical (CD14+CD16++, right) monocyte subsets in COVID-19 patients **(A)** and healthy controls **(B)** after antigen stimulation with *C. albicans* and HKLM. Values represent percentages of total monocytes. Boxes indicate median and interquartile range; whiskers represent minimum and maximum. Dots represent single values. Different incubation conditions were compared using One Way ANOVA (*P< .05, **P< .01). COV, COVID-19 (n=11); HC, healthy controls (n=9).
